# Derivation of marker gene signatures from human skin and their use in the interpretation of the transcriptional changes associated with dermatological disorders

**DOI:** 10.1002/path.4864

**Published:** 2017-02-24

**Authors:** Barbara B Shih, Ajit J Nirmal, Denis J Headon, Arne N Akbar, Neil A Mabbott, Tom C Freeman

**Affiliations:** ^1^ The Roslin Institute and Royal (Dick) School of Veterinary StudiesUniversity of Edinburgh, Easter BushMidlothianEdinburghEH25 9RGUK; ^2^ Division of Infection and ImmunityUniversity College London90 Gower StreetLondonWC1E 6BTUK

**Keywords:** transcriptomics, gene expression, skin, sebaceous gland, apocrine gland, sweat gland, psoriasis

## Abstract

Numerous studies have explored the altered transcriptional landscape associated with skin diseases to understand the nature of these disorders. However, data interpretation represents a significant challenge due to a lack of good maker sets for many of the specialized cell types that make up this tissue, whose composition may fundamentally alter during disease. Here we have sought to derive expression signatures that define the various cell types and structures that make up human skin, and demonstrate how they can be used to aid the interpretation of transcriptomic data derived from this organ. Two large normal skin transcriptomic datasets were identified, one RNA‐seq (n = 578), the other microarray (n = 165), quality controlled and subjected separately to network‐based analyses to identify clusters of robustly co‐expressed genes. The biological significance of these clusters was then assigned using a combination of bioinformatics analyses, literature, and expert review. After cross comparison between analyses, 20 gene signatures were defined. These included expression signatures for hair follicles, glands (sebaceous, sweat, apocrine), keratinocytes, melanocytes, endothelia, muscle, adipocytes, immune cells, and a number of pathway systems. Collectively, we have named this resource SkinSig. SkinSig was then used in the analysis of transcriptomic datasets for 18 skin conditions, providing in‐context interpretation of these data. For instance, conventional analysis has shown there to be a decrease in keratinization and fatty metabolism with age; we more accurately define these changes to be due to loss of hair follicles and sebaceous glands. SkinSig also highlighted the over‐/under‐representation of various cell types in skin diseases, reflecting an influx in immune cells in inflammatory disorders and a relative reduction in other cell types. Overall, our analyses demonstrate the value of this new resource in defining the functional profile of skin cell types and appendages, and in improving the interpretation of disease data. © 2016 The Authors. *The Journal of Pathology* published by John Wiley & Sons Ltd on behalf of Pathological Society of Great Britain and Ireland.

## Introduction

Disorders of the skin are ranked as the fourth leading cause of non‐fatal disease burden 1, and consequently there is significant interest in better understanding the biology of this organ and its dysregulation. Modern platforms allow the quantitative analysis of the complete set of transcripts expressed in a given sample. These technologies have been used to characterize the transcriptome of normal skin 2 and to determine how this is altered in certain skin diseases 3. However, interpretation of such data remains a significant challenge. During disease, the biology and the cellular composition of the skin may change significantly: for example, due to the influx of immune cells. In addition, when comparing samples gathered from different sites or by different methods, the cellular composition of individual samples may vary significantly. Such differences will be reflected in the transcriptional profile of the sample, but at present, many of the genes expressed in skin appendages (e.g. hair follicles and eccrine, apocrine, and sebaceous glands) or cell types are largely uncharacterized and therefore changes in their abundance may be difficult to interpret. If we knew the genes specifically expressed by cells found in the skin, we could use them to assess their relative abundance in samples, thereby allowing us to better interpret observed changes in transcriptomic data derived from this organ.

In an effort to address this problem, Swindell *et al* 4 used publicly available microarray data from isolated cell populations to define 24 ‘cell‐type specific’ signatures and used them to aid the interpretation of genes differentially expressed in psoriasis, and subsequently in multiple skin disorders 3. Although comprehensive gene marker sets for numerous immune cell subtypes were obtained, those for many types of skin appendages and cell types were not. Li *et al* 5 used a weighted gene co‐expression network analysis approach 6 to derive co‐expression clusters from RNA‐seq data of normal and psoriasis skin biopsies. The gene clusters reported were mostly large in size, ranging from 32 to 5427 genes, with several cell types overrepresented in multiple modules 5. Gene signatures for skin appendages and some cell types were also lacking in this study, as the annotation of these co‐expression clusters was predominately based on those reported by Swindell *et al* 4.

We therefore set out to identify sets of genes diagnostic of the many cell types present in human skin. Our approach is based on the fact that the expression level of a gene expressed specifically in a particular cell type or skin appendage will vary according to its abundance in a given sample. When measured across multiple samples, any other genes expressed in a similar manner will be observed to be co‐expressed. When correlation networks are constructed from transcriptomic data 7, 8, 9, groups of co‐expressed genes form highly connected cliques within the network, which can then be defined by network clustering algorithms 10. Taking advantage of this and the subtle variation in cellular composition between human skin samples (either intrinsic to a sample or due to variation in sampling), cell‐specific gene signatures may be extrapolated without the need to physically isolate specific cell types or skin appendages.

Here, we describe the derivation of 20 highly conserved gene expression signatures, collectively named *SkinSig*, for skin appendages, cells, and processes present in human skin. This resource aids the interpretation of transcriptomic data derived from human skin, allowing the cellular composition of samples to be explored. Furthermore, these signatures enable the pathological and physiological changes associated with skin conditions, disease subtypes or therapeutic interventions to be characterized.

## Materials and methods

### Data acquisition, quality control, and processing

The RNA‐seq dataset (mapped to gene level and RPKM‐normalized) was obtained from the GTEx project (http://www.gtexportal.org) 2, which at the time of download consisted of 607 post‐mortem skin samples. Further details on the dataset are provided in the supplementary material, Table S1. Quality control (QC) for the RNA‐seq dataset involved sample–sample correlation analyses, performed using the analysis software Miru (Kajeka Ltd, Edinburgh, UK). The topology of the sample–sample correlation was examined against the sample metadata, which identified major discrepancies between earlier and later RNA‐seq batches. Removal of early batches of data (LCSET‐1156 to LCSET‐1480) left a total of 578 samples, comprising 250 suprapubic and 328 lower leg samples.

A microarray dataset of normal skin was generated by combining the normal samples from two large psoriasis studies (GSE13355 and GSE30999) performed on the Affymetrix U133 plus 2.0 array and downloaded from the Gene Expression Omnibus (GEO) 11, 12, 13. Further details on these datasets are provided in the supplementary material, Table S1. Both QC and batch correction were carried out on the microarray dataset. Samples detected as outliers by the metrics report of arrayQualityMetrics 14 were excluded from further analysis. Samples passing QC were normalized using frozen robust multi‐array analysis 15 within study, followed by intra‐ and inter‐study batch correction using ComBat (sva, surrogate variable analysis; Bioconductor package) 16. Normalized data were loaded into Miru for sample–sample correlation analysis. Following the removal of outliers and all samples from psoriatic psoriasis skin, 165 normal (healthy subjects or non‐lesional skin from psoriasis subjects) samples were available for downstream analyses.

### Co‐expression network analysis and cluster annotation for individual datasets

Of the 56 318 transcripts (genes defined in GENCODE v19) in the original RNA‐seq dataset, 24 128 transcripts were expressed in normal skin (>1 RPKM in at least one sample). The filtered dataset of skin‐expressed transcripts was loaded into Miru, with a gene–gene Pearson correlation threshold (*r*) set at *r* ≥ 0.73. A similar process was repeated for the microarray dataset at probe level with a correlation threshold of *r* ≥ 0.66. The Markov clustering (MCL) algorithm 10 was used with an inflation value of 2.2 for identifying co‐expression clusters.

In order to identify the functional relevance of transcript clusters, we used a combination of bioinformatics tools, literature review, as well as similarity to previously defined co‐expression clusters 17, 18. Each co‐expression cluster was examined using a number of bioinformatics tools, including gene ontology (GO) annotation enrichment analysis (http://pantherdb.org; Gene Ontology database release 2016‐04‐23), pathway inspection (Reactome, http://www.reactome.org; KEGG, http://www.genome.jp/kegg), and protein localization (Human Protein Atlas, http://www.proteinatlas.org). In addition, co‐expression signatures from previous studies were manually compared with clusters derived from skin, allowing the naming of some of the signatures. Clusters without GO enrichment or without similarity to previously reported co‐expression signatures were further investigated by checking individual genes against the literature and the phenotypes reported for knockout mice.

### SkinSig derivation

In order to compare across analyses, the RNA‐seq and the microarray data were mapped to HGNC gene symbols (Ensembl BioMart, release 84), which were used as common identifiers. Where more than one microarray probe targeted a given symbol, the probe set with the highest median absolute deviation for signal intensity was used. Both datasets were further filtered to include only genes common to both platforms and with an expression greater than 1 RPKM in at least one sample in the RNA‐seq dataset. A total of 15 736 transcripts met these criteria.

Correlation networks were created from the common‐symbol filtered datasets at *r* ≥ 0.66 for the microarray dataset and *r* ≥ 0.73 for the RNA‐seq dataset. Network‐derived clusters from each study were individually annotated with the assistance of signature tracks imported from the network analysis of the full transcript/probe set analyses. Genes with the same annotation for both studies were used to construct *SkinSig*, together with three study‐specific annotations (circadian clock, skeletal muscle, and apocrine gland).

### Application of SkinSig to gene expression data from skin conditions

The utilities of *SkinSig* in analysing gene expression data were demonstrated using existing microarray or RNA‐seq datasets from a variety of skin conditions. The combined dataset originally organized and normalized by Inkeles *et al* 3 was included, alongside six additional datasets identified in GEO, covering a total of 18 skin conditions. The dataset from ref 3 comprised microarray data from 15 skin conditions and normal skin, all based on the Affymetrix U133 plus 2.0 array platform. The six additional studies used a variety of platforms, covering six different skin conditions, three of which were not investigated in ref 3. An overview on each dataset is provided in the supplementary material, Table S1. In ref 3, the control group comprised normal skin samples from multiple studies (supplementary material, Table S1). For all other datasets, the test groups (i.e. skin condition) were compared against the control groups within the same study. Complex study designs were simplified accordingly (supplementary material, Table S1). Ageing gene expression data from Glass *et al* 19 were analysed by comparing each age group (50–60, 60–70, and > 70 years old) with the youngest group (≤50 years).

For each dataset, the geometric means were used to average across multiple probes for the same HGNC symbol. The expression level of each signature was calculated as an average of all genes within a signature (transformed to log_2_ prior to averaging). The log fold changes between these values from the pairing test and control groups were used to plot a heatmap, thereby allowing comparison across the different skin conditions. Positive fold change reflects overrepresentation of the signature in the test group, i.e. a particular skin condition. Details on each test–control pairing are described in the supplementary material, Table S1. Rotation gene set test (ROAST) (limma, linear models for microarray data; Bioconductor package) 20 was used to assess the statistical difference in expression between the control and the test groups, treating each gene within a signature as a separate measurement. Signatures were considered to be significantly altered when the false discovery rates (FDRs) ≤ 0.01 and ≥ 80% genes changed in the same direction (increased or decreased).

The effect of psoriasis on the keratinocyte differentiation signature was examined using quality‐controlled raw data from GSE13355 and GSE30999 and processed data for GSE54456 (supplementary material, Table S1). Co‐expression network analyses were carried out on the keratinocyte differentiation signature for each study, with only normal skin, only psoriatic skin or all samples. By using the MCL algorithm (inflation value = 2.2) on the co‐expression network for all samples from GSE13355, two subgroups of the keratinocyte differentiation signature were identified. ROAST was used to determine whether the keratinocyte differentiation subgroups were significantly differentially expressed. An FDR ≤ 0.01 was considered to be significant.

The study only involved publicly available de‐identified data; ethical approval was not required in the country/region in which the study was carried out (Scotland, UK).

## Results

### Network analysis of transcriptomic data from normal skin

Two datasets representing large collections of normal skin biopsies were selected (GSE13355 and GSE30999) 11, 12, 13. These comprised microarray data from healthy subjects or non‐lesional skin from psoriasis subjects of mixed anatomical origin (*n =* 165). In addition, a collection of RNA‐seq data from biopsies of post‐mortem normal skin from lower leg or suprapubic regions (*n =* 578) was obtained from the Genotype‐Tissue Expression (GTEx) project 2.

We interrogated these datasets using co‐expression network analysis 18, 21. Here, subtle differences between the normal skin biopsies result in groups of co‐expressed genes forming highly connected cliques within the network's overall topology. This method relies on characterizing groups of co‐expressed genes, rather than conventional analysis of statistically significant differences between pre‐determined sample groups. Co‐expression networks were independently constructed for the microarray and RNA‐seq datasets.

Following QC and batch correction, both the RNA‐seq and the microarray datasets showed little overall variation between samples, suggesting little variation attributable to technical or biological factors (supplementary material, Figure S1A, B). Although the microarray and RNA‐seq datasets differed in a number of respects (analysis platform, sampling site, live versus dead donors, etc.), the median expression levels for the majority of genes expressed across the two studies were consistent (supplementary material, Figure S1C). However, genes with little expression (<100 signal intensity, microarray; < 1 RPKM, RNA‐seq) demonstrated a non‐linear relationship between the two platforms. Saturation of microarray probes and a non‐linear signal response for highly expressed genes is a known limitation of microarray analyses, and here, the signal intensity plateaued at a signal intensity value of approximately 16 000 (supplementary material, Figure S1C). A small number of genes are observed to be very highly expressed in RNA‐seq data relative to others (supplementary material, Figure S1D).

**Figure 1:**
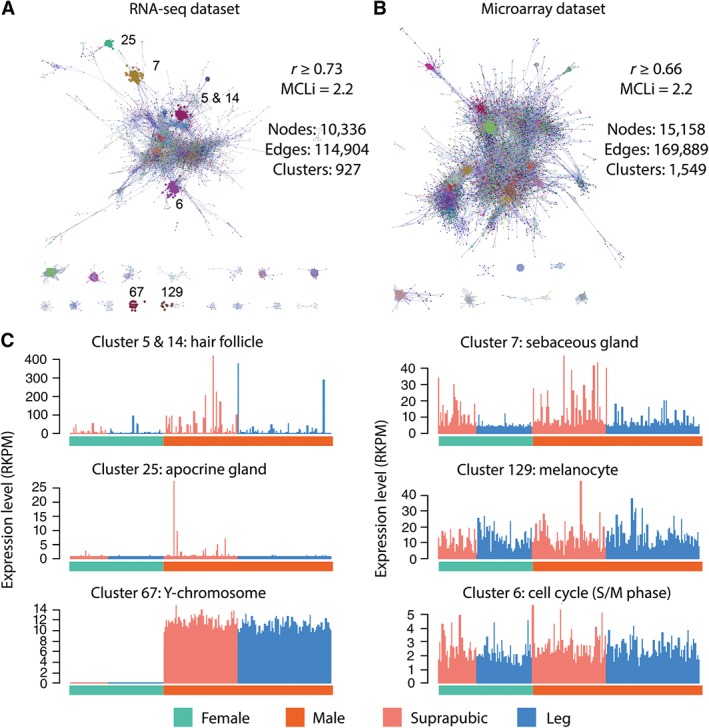
Network analysis of the RNA‐seq and microarray datasets. The IDs for some of the clusters are noted on the co‐expression network for (A) the RNA‐seq and (B) microarray datasets. Nodes are coloured according to cluster membership. Nodes represent genes or transcripts, and edges correlations between them above the Pearson correlation threshold value. (C) The average expression profile for a number of the clusters (highlighted in B) found in the RNA‐seq dataset, in some cases corresponding to the expected trends across gender and sampling location (suprapubic or leg) or gender.

Correlation analysis is based on the analysis of statistically improbable relationships (Figure S2) which are used in the generation of co‐expression networks. Use of the MCL algorithm 10 enables these networks to be divided into gene clusters that share a similar expression pattern across the dataset. The optimal Pearson correlation coefficient *r* value used to construct each network was determined empirically. At an *r* value of 0.73, the RNA‐seq dataset yielded a co‐expression network which was composed of 10 336 nodes (genes), connected by 114 904 edges, and contained 927 clusters (Figure 1A). The majority of the clusters were small; only 24 clusters had more than 50 genes, and 123 clusters had more than ten genes. Similar analyses were carried out on the microarray dataset at *r* ≥ 0.66, where the co‐expression network was composed of 15 158 nodes, 169 889 edges, and 1549 clusters (Figure 1B). The gene clusters derived from each dataset were mined extensively to understand their gene content. The significance of some clusters was easy to explain as their contents shared a high degree of similarity to those observed previously and/or were enriched in genes with informative GO annotations. Other gene clusters were less easy to interpret and required manual curation and expert review. The full cluster list, their gene composition, and functional annotations are provided in the supplementary material, Table S2.

The RNA‐seq dataset contained several gene clusters that appeared to be derived from the ‘contamination’ of samples from explicable sources, including skeletal muscle (Cluster 12), neurones (Cluster 43), and glial cells (Cluster 83) (see supplementary material, Table S2). Three clusters of genes were unexpected and inexplicable in skin samples; Cluster 1 (spermatids, expression predominately associated with male suprapubic samples) contained many genes involved in spermatogenesis, Cluster 29 (pancreas) contained genes encoding pancreatic enzymes (such as pancreatic lipases, proteases, and insulin), whereas Cluster 97 (stomach mucosa) contained genes encoding gastrokine, gastric lipase, and pepsinogens (see supplementary material, Table S2). However, many of the remaining clusters observed in the RNA‐seq dataset were found to show significant overlap with gene clusters present in the microarray dataset (see below). Exceptions included a cluster of genes exclusively expressed in the suprapubic samples which contained several apocrine gland markers, e.g. ABCC11 and ACSM1 (Figure 1C), reflecting the restricted presence of these glands to pubic and axillary skin regions 22. Conversely, a small cluster observed only in the microarray dataset consisted exclusively of circadian clock‐associated genes, such as PER1 and PER3, and may reflect the difference in the time of sampling or the use of post‐mortem samples in the RNA‐seq dataset.

### Derivation conserved skin gene signatures (SkinSig)

**Figure 2:**
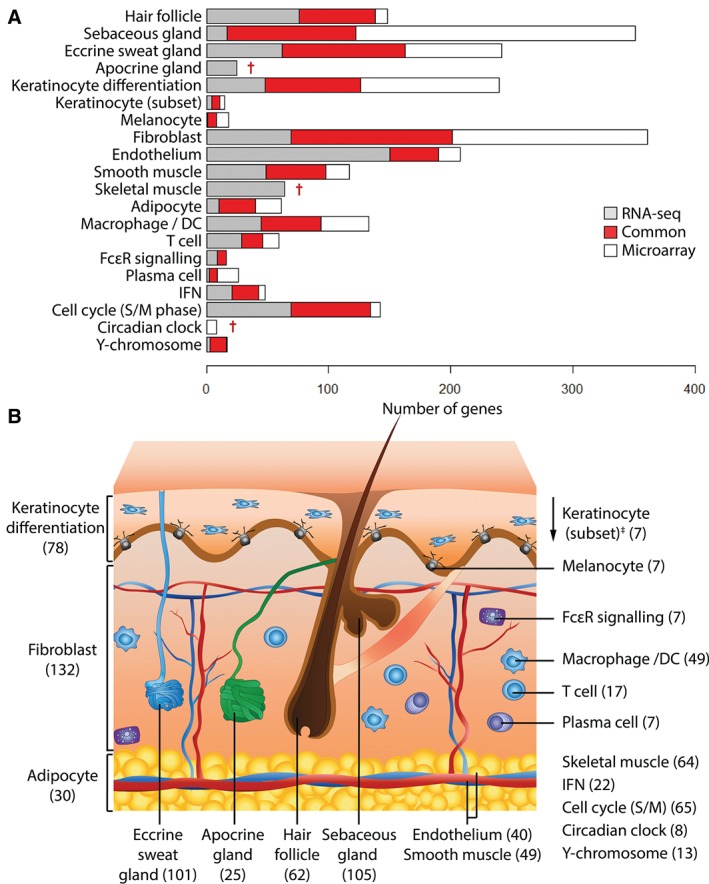
Cross validation of signatures between the RNA‐seq and microarray datasets. (A) When gene clusters derived individually from the two datasets were compared, a portion of the genes was common to both analyses (red). These genes form the basis of SkinSig. Some clusters consisted of more genes in the RNA‐seq dataset (grey) than the microarray dataset (white) and vice versa. (B) A schematic diagram of skin illustrates the number of marker genes for each signature. ^†^Study‐specific signatures. ^‡^The keratinocyte (subset) is a small group of highly co‐expressed genes but whose expression is independent of the keratinocyte differentiation signature genes.

We identified 17 signatures with overlapping gene membership in both the RNA‐seq and the microarray datasets (Figure 2A). These consisted of clusters identified as being derived from the majority of the appendages and cell types present in skin, as well as clusters of genes associated with core biological pathways, such as the cell cycle (Table 1 and supplementary material, Table S3). Within each signature, the overlap in gene membership in the RNA‐seq and the microarray datasets was 59 ± 18% and 63 ± 22% (mean ± SD), respectively (Figure 2A and Table 1). In order to define a robust set of marker genes, only those present in the same signature in both datasets were included in the final signature lists. The overlapping gene sets should be considered a highly conservative list of transcripts associated with skin appendages, cells, and pathways.

**Table 1:** Overlap between annotation made on the co‐expression clusters derived from network analysis of the RNA‐seq and microarray datasets

**Cluster annotation**	**RNA‐seq‐specific**	**Microarray‐specific**	*SkinSig* (No. of genes)	% Common (relative to the RNA‐seq dataset)	% Common (relative to the microarray dataset)
Hair follicle	76	10	62	45%	86%
Sebaceous gland	17	229	105	86%	31%
Eccrine sweat gland	62	79	101	62%	56%
Apocrine gland	25	0	25	Dataset‐specific	
Keratinocyte differentiation	48	114	78	62%	41%
Keratinocyte (subset)	4	4	7	64%	64%
Melanocyte	1	10	7	88%	41%
Fibroblast	69	160	132	66%	45%
Endothelium	150	18	40	21%	69%
Smooth muscle	49	19	49	50%	72%
Skeletal muscle	64	0	64	Dataset‐specific	
Adipocyte	10	21	30	75%	59%
Macrophage/DC	45	39	49	52%	56%
T cell	29	13	17	37%	57%
FcϵR signalling	9	0	7	44%	100%
Plasma cell	2	17	7	78%	29%
IFN	21	5	22	51%	81%
Cell cycle (S/M)	69	8	65	49%	89%
Circadian clock	0	8	8	Dataset‐specific	
Y‐chromosome	3	1	13	81%	93%

In addition to these 17 signatures, three additional signatures that were only observed in one of the two datasets were also included: circadian clock (microarray only), apocrine gland, and skeletal muscle (RNA‐seq only) (Figure 2B and Table 1). Apocrine glands are only found in certain skin regions, such as external genitalia (suprapubic samples) present only in the RNA‐seq samples. Skeletal muscle contamination, presumably due to biopsy depth, was also only evident in the RNA‐seq dataset. We have collectively named these gene signatures *SkinSig*.

**Figure 3:**
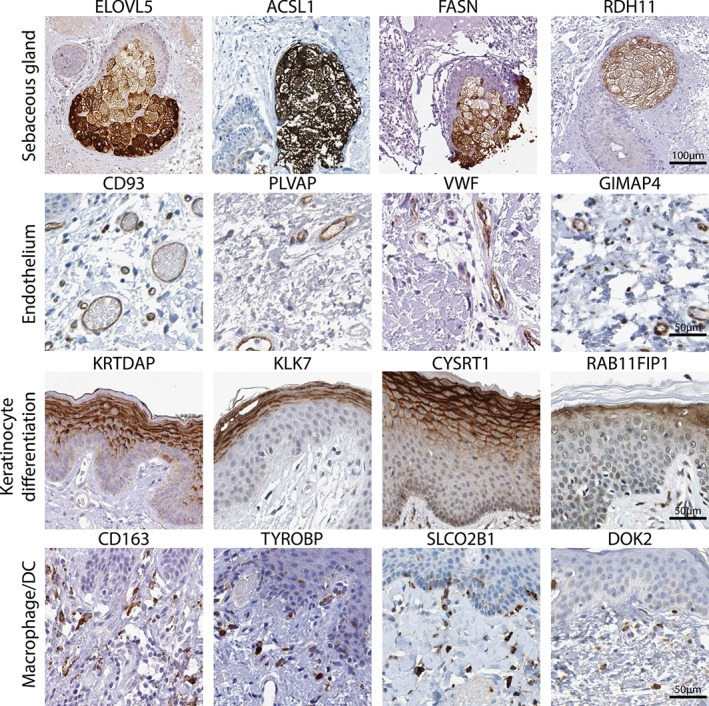
Examples of immunohistochemical staining of proteins encoded by SkinSig marker genes. Localization of a number of the proteins encoded by a selection of SkinSig marker genes, verifying the assignment given. Reprinted from the Human Protein Atlas database 23, with permission.

GO enrichment terms and key marker genes present in each signature are detailed in the supplementary material, Table S3. However, GO enrichment analysis did not help with the functional assignation of some signatures, including sebaceous gland, apocrine gland, eccrine sweat gland, keratinocyte (subset), and Y‐chromosome. The justification and relevant literature supporting the functional annotation of these signatures are discussed in the supplementary material, Supplementary discussion. Our analyses obtained several novel marker gene signatures defined here for the first time. These include signatures for hair follicles, sebaceous glands, eccrine sweat glands, apocrine glands, and melanocytes. The localization of proteins encoded by some of these genes is independently confirmed in immunohistochemistry images derived from the Human Protein Atlas 23 (Figure 3).

### Application of SkinSig to the interpretation of gene expression data

**Figure 4:**
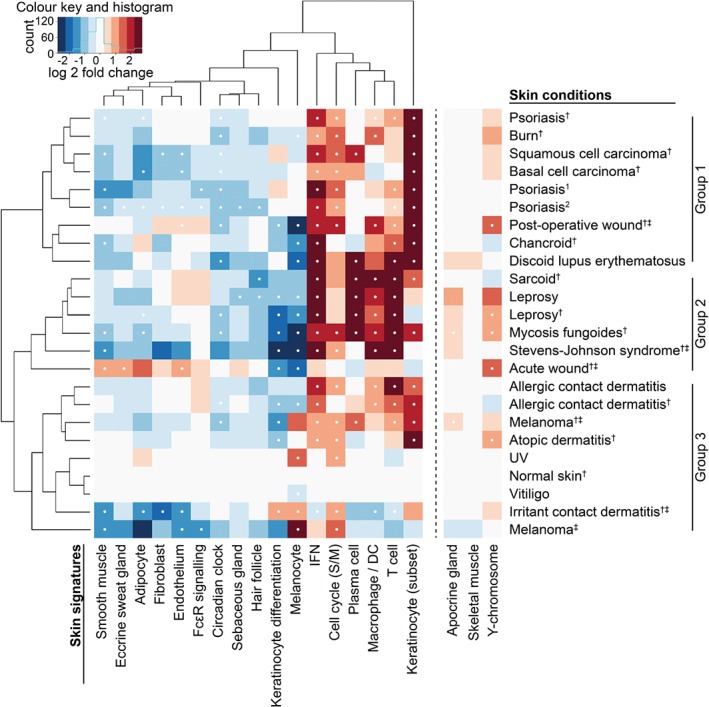
Skin signatures applied in analysis for 18 skin conditions. The heatmap and dendrogram were derived from the log_2_ fold change for each skin condition (test versus control group) for the 17 skin signatures on the left‐hand side of the dashed line. Expression of the remaining three signatures, apocrine gland, skeletal muscle, and Y‐chromosome, is highly dependent on sample properties unrelated to the condition (such as gender, contamination or sampling sites) and was therefore not included in the clustering. Full details of the datasets can be found in the supplementary material, Table S1. Significantly altered signatures (FDR ≤ 0.01 and ≥ 80% of the genes altered in the same direction) are indicated with a white dot. Due to the stated criteria for a comparison to be considered significant, there are instances where the average expression appears strongly dysregulated but is not considered significant; in these cases, it may be that FDR ≤ 0.01 but only < 80% of the signature is altered in the same direction. ^†^Data for each skin condition derived from ref 3 were compared against the data for the same group of normal skin derived from multiple studies. ^‡^Sampling designs for these studies may introduce an artificially‐altered balance between cell populations (such as complete removal of epidermis) in the test group, but not the control group. Further details may be found in the supplementary material, Table S1.

To validate and test the utility of *SkinSig*, we gathered transcriptomic data from studies of 18 different skin conditions (pathological or physiologically altered). These included the combined dataset reported by Inkeles *et al* 3 and a further six datasets downloaded from GEO (see supplementary material, Table S1). For each skin condition, the fold change in expression level of all genes within each *SkinSig* gene signature was calculated and compared with control samples (supplementary material, Table S1). Each skin condition had a specific profile of altered gene expression of the *SkinSig* signatures. Hierarchical clustering of the signatures (excluding skeletal muscle, Y‐chromosome, and apocrine gland) based on these analyses revealed three main groupings of skin conditions (Figure 4).

**Figure 5:**
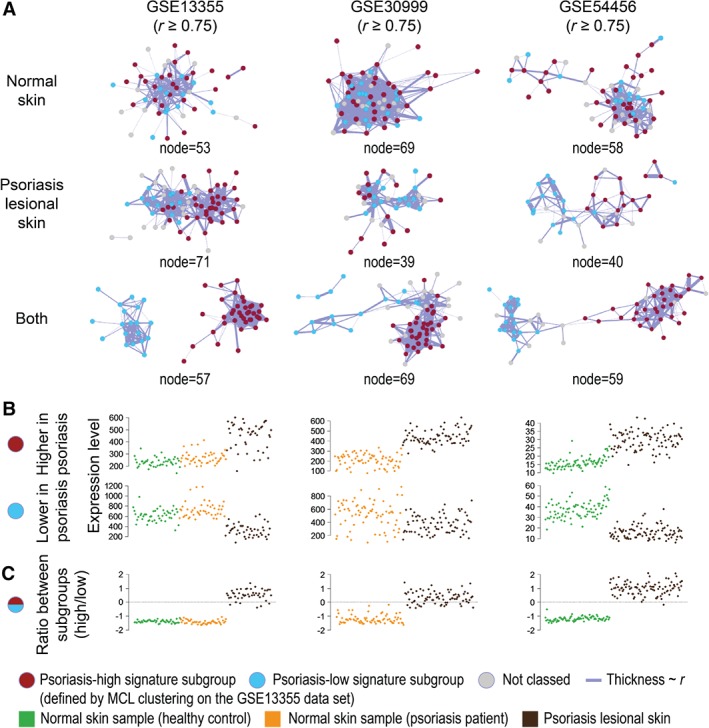
Alteration in the keratinocyte differentiation signature in psoriasis. Co‐expression networks were constructed using only genes in the keratinocyte differentiation signature for psoriasis studies, GSE13355 (left), GSE30999 (middle), and GSE54456 (right). (A) Using the MCL algorithm on the network graph for the full dataset of GSE13355, the signature was split into two subgroups, one up‐regulated (red) and one down‐regulated (blue) in psoriasis. This clustering has been overlaid on the networks derived from the other studies. The separation of the keratinocyte differentiation signature subgroups is dependent on the sample types included in the network analysis: normal skin only (top), psoriatic lesions only (middle), and both sample types (bottom). (B) The average expression for all genes within each subgroup for each sample. (C) The log_2_ ratios between the expression of the two subgroups are generally lower than zero in the control samples. On the other hand, these ratios are higher and more variable in between psoriatic samples, perhaps reflecting disease severity.

We also analysed an additional dataset comprising normal and psoriatic skin (GSE13355; *n* = 46 external normal skin; *n* = 46 patient‐matched normal skin; *n* = 45 patient‐matched psoriatic skin) 11. The keratinocyte differentiation signature was found to split into two subgroups of genes which were significantly up‐ (38 genes) and down‐regulated (19 genes) in psoriasis (FDR ≤ 0.01) (Figure 5A). When these two subgroups of genes were applied to two additional psoriasis datasets (GSE30999 and GSE54456), we observed a similar trend (Figure 5B), demonstrating that this loss of co‐expression between the two subgroups was replicable across independent studies.

**Figure 6:**
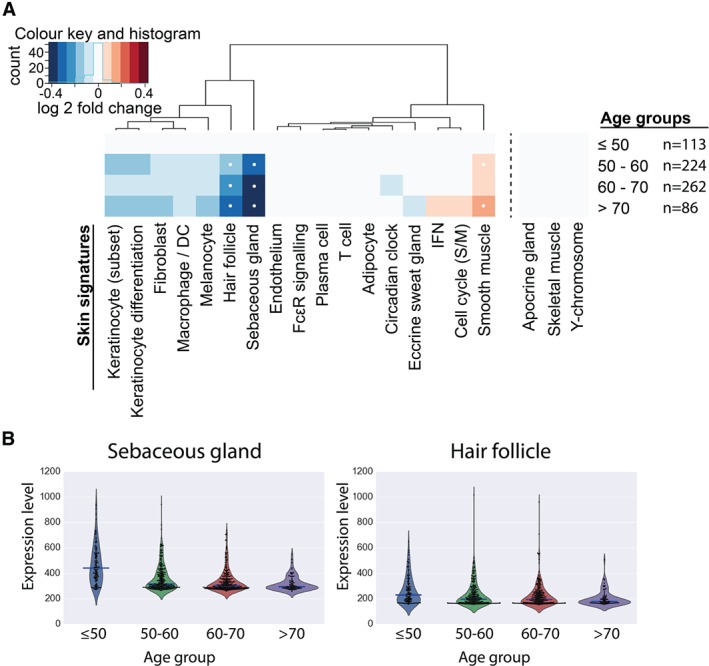
Effects of ageing on SkinSig. (A) The heatmap illustrates the changes in SkinSig during ageing by comparing each age group with the youngest age group (≤50 years old), with comparisons yielding statistical significance (FDR ≤ 0.01 and ≥ 80% genes altered in the same direction) highlighted with a white dot. The fold change was calculated from the log_2_ mean expression for genes present in each signature. Rotation gene set tests were employed for statistical analysis. (B) The violin plots show the average expression for the hair follicle and sebaceous gland signatures for each individual. These plots show the change in distribution across age groups. The median expression value for each age group is indicated by a blue bar.

Lastly, we applied *SkinSig* to a collection of data from ageing human skin (infra‐umbilical skin from female twins) 19. *SkinSig* revealed significant changes (FDR ≤ 0.01) to gene signatures associated with hair follicles and sebaceous gland in ageing skin (Figure 6). Approximately 44–62% of the content of these signatures was reported to be differentially expressed in ageing skin in the original study 19 (supplementary material, Table S4).

## Discussion

Here, co‐expression network analysis has been used to interrogate transcriptomic data derived from normal human skin. Using this approach, we derived 20 co‐expression signatures that characterize the function‐specific profile of many cell types and appendages present in the skin. The most conserved of these co‐expressed genes across the two primary datasets used for this study have been named *SkinSig*. Evidence supporting these gene set annotations is available in Table S3 and the Supplementary discussion (supplementary material), and an expanded list of annotated gene clusters derived from these studies, including the analysis of the data from the MuTHER study 19, is presented in Table S2 (supplementary material).

The utility of *SkinSig* was demonstrated by using the signatures to describe the transcriptional changes associated with 18 skin conditions and ageing. *SkinSig* broadly separated the skin conditions into three groups (Figure 4). Group 1 included psoriasis, discoid lupus erythematosus, squamous cell carcinoma, and basal cell carcinoma (Figure 4). Each of these presents epidermal changes such as hyperkeratosis and epidermal hyperproliferation 24, 25, and was characterized by marked increases (FDR ≤ 0.01) in the keratinocyte (subset) and IFN signatures. Most conditions within this group also demonstrated a significant increase in the cell cycle signature (FDR ≤ 0.01). This group also included burns, post‐operative wounds, and chancroid, each of which was associated with wound healing or ulceration.

Group 2 was characterized by strongly up‐regulated T‐cell, IFN, and macrophage/dendritic cell (DC) signatures (FDR ≤ 0.01 for all but acute wound), together with down‐regulated keratinocyte differentiation and melanocyte signatures (FDR ≤ 0.01 for all but sarcoid) (Figure 4). The overall *SkinSig* expression pattern for groups 1 and 2 suggested that the relative contribution of other cell types to the transcriptional profiles of these samples was diluted: their signatures appear to be down‐regulated by the influx of immune cells and associated up‐regulation of immune response genes.

Group 3 generally showed less transcriptional perturbation relative to normal skin. UV‐challenged skin and vitiligo were most similar to normal skin, with the former showing an increase and the latter a decrease in the expression of the melanocyte signature (Figure 4). UV exposure would be expected to induce melanocyte proliferation 26, while vitiligo involves melanocyte loss from patches of skin 27. Other disorders in this group showed changes in the *SkinSig* signatures consistent with the known pathology of those conditions, such as the increased expression of the T‐cell signature in allergic contact dermatitis 28.

This study highlights the need to consider the sampling protocols used when interpreting transcriptomic data (Figure 4 and supplementary material, Table S1). In the case of the acute and post‐operative wound samples, the epidermis was removed prior to analysis 29, whereas the dermis was removed from the irritant contact dermatitis samples 30, and only blister fluid was used in the analysis for the Stevens–Johnson syndrome dataset 31. The complete removal of epidermis in the acute wound dataset, and the use of only epidermis for the irritant contact dermatitis dataset, may explain the unusual pattern within the heatmap for these two skin conditions; the former shows underrepresentation of epidermal cell types and overrepresentation of dermal cell types, whilst the reverse is true for the latter (Figure 4). Macro‐dissection or enrichment in tumour cell populations may likewise result in reduced resident skin cell diversity and abundance in samples such as those from melanoma and mycosis fungoides 32, 33.

Intriguingly, the keratinocyte differentiation signature did not appear to be up‐regulated in any of the skin conditions, including psoriasis. Upon closer examination of the expression of this signature in three independent psoriasis datasets, two subgroups of the keratinocyte differentiation signature genes were identified, and the ratio between the expression levels of these two subgroups was altered in psoriatic skin (Figure 5C). Higher variation of this ratio was also noted across the psoriatic samples, which may reflect the magnitude of the disease severity (Figure 5C). Within the keratinocyte differentiation signature subgroup that is down‐regulated in psoriasis, several genes, such as *LCE1B*, *LCE2B*, *FLG2*, and *LOR*, are known to be associated with the terminally differentiated keratinocytes (cornecytes) that make up the stratum corneum. Furthermore, *LCE3B* and *LCE3C* deletions have been identified as risk factors for psoriasis in multiple ethnic groups 34. Several genes known to be expressed in suprabasal keratinocytes were present within the keratinocyte differentiation signature subgroup up‐regulated in psoriasis. The altered expression profile of these genes most likely reflects the hyperproliferation of the epidermis that is associated with psoriasis 35. In short, genes in these subgroups of the keratinocyte differentiation signature were expressed at a similar ratio across normal skin, but become uncoordinated in psoriatic lesions. Instead of considering lists of dysregulated genes, this co‐expression approach with *SkinSig* allows the recognition of a disrupted system by defining the dynamics between genes in a physiologically normal state.

Glass *et al* 19 have reported a decrease in the expression of genes during skin ageing associated with keratinization and fatty acid metabolism. Using *SkinSig*, these changes may more accurately be described as a reduction in the number or functional activity of sebaceous glands and hair follicles with age (Figure 6). These observations are consistent with the rapid decline in scalp hair coverage in women over 45 years old 36. The reduction in the sebaceous gland signature is also consistent with previous reports showing gradually decreased activity of this gland after menopause 37. A small increase in the expression of smooth muscle signature is also seen in the age groups 50–60 and above 70 years (Figure 6); migration and accumulation of vascular smooth muscle cells into the tunica intima have been implicated in ageing 38.

In summary, we have defined a set of marker genes, collectively named *SkinSig*, which comprise a useful resource of gene signatures derived from skin appendages, cell types, and pathways present in normal human skin. *SkinSig* not only includes potential new marker genes for skin‐resident cell types and processes, but can also be used to interrogate gene expression data derived from whole human skin. Importantly, *SkinSig* can be used to obtain novel insights into the physiological and pathological changes that occur in the skin.

## Author contributions statement

BS carried out the collection, analysis, and interpretation of data, as well as compilation of the figures and tables. AN and DH contributed to data analysis and interpretation. NA, NM, and TF contributed to the study design. NM and TF contributed equally. All authors were involved in writing the paper and had final approval of the submitted and published versions.

**SUPPLEMENTARY MATERIAL ONLINE**

**Supplementary discussion.** Further discussion on other gene clusters of interest and justifications for the assignation of annotation of co‐expression signatures without a significant or relevant gene ontology term

**Supplementary figure and table legends**

**Figure S1.** Sample–sample correlation and signal comparisons between the RNA‐seq and microarray datasets

**Figure S2.** Pearson correlation thresholds in randomized and original data

**Table S1.** Details on the datasets used in this study

**Table S2.** *SkinSig* and co‐expression signatures derived from the analysis of different datasets derived from normal human skin.

**Table S3.** Gene ontology enrichment and example genes for each signature

**Table S4.** Comparison between *SkinSig* and genes found to be significantly altered during ageing

## Supporting information


**Supplementary discussion.** Further discussion on other gene clusters of interest and justifications for the assignation of annotation of co‐expression signatures without a significant or relevant gene ontology term

**Supplementary figure and table legends**

**Figure S1 Sample–sample correlation and signal comparisons between the RNA‐seq and microarray datasets. (A)** Sample–sample correlation plots of data used in these studies, using the maximum Pearson correlation coefficient threshold that still retained all samples for the RNA‐seq (r ≥ 0.93) and **(B)** the microarray (r ≥ 0.97) datasets. There is minimal sample separation due to the main sample attribute, i.e. study (microarray) or site of sampling (RNA‐seq). **(C)** Log‐scale plot of median expression values, showing that expression levels for the majority of genes are positively correlated in the two datasets. **(D)** Of the top ten genes with the highest expression for the microarray (red) and RNA‐seq (blue) datasets, there were only two genes in common (RPS18 and KRT14). A higher dynamic range was also observed in the RNA‐seq dataset.

**Figure S2 Pearson correlation thresholds in randomized and original data. (A)** When expression values for each gene were randomized across the samples of the RNA‐seq dataset, only 95 pairing (edges) were observed at a threshold used in this analysis (r ≥ 0.73), whilst the untransformed data yielded 87 121 edges. Taking into account that a total of 123 802 980 calculations were made for every possible combination of gene–gene Pearson correlation analysis, the frequency of a pair of genes reaching the r threshold is 7.7 × 10^−7^ (blue), compared with a frequency of 7.0 × 10^−4^ for non‐random correlations observed in the actual dataset (red). This supports the notion that the vast majority of relationships used to build the network analysed here are non‐random. **(B)** Similar analysis was done to the microarray dataset. The randomized version of the dataset shows a frequency of 1 × 10^−8^ for a random correlation (blue) to occur at r ≥ 0.66, compared with a frequency of 6.7 × 10^−4^ observed in the actual dataset (red).

**Table S1 Details on the datasets used in this study** The subject details, experimental design, data source, and comparisons for all datasets included in this study are listed in Table S1. This includes the three primary data datasets, 22 validation datasets for skin conditions, three psoriasis datasets for integrating the keratinocyte differentiation signature, and one ageing dataset.

**Table S2 SkinSig and co‐expression signatures derived from the analysis of different datasets derived from normal human skin** The supplementary file includes the co‐expression signatures for all network analysis, including the individual full original datasets, gene‐symbol restricted datasets, and *SkinSig*.

**Table S3 Gene ontology enrichment and example genes for each signature** In addition to highlighting a number of key genes and significant gene ontology (GO) enrichment result for each *SkinSig*, the supplementary file also includes the full set results from gene ontology enrichment.

**Table S4 Comparison between SkinSig and genes found to be significantly altered during ageing** Genes reported to change in their expression during ageing are compared with *SkinSig*, showing a high percentage of the sebaceous gland and hair signatures being reported to alter with ageing by Glass *et al* [19].
