# Supplementary material for: Derivation of marker gene signatures from human skin and their use in the interpretation of the transcriptional changes associated with dermatological disorders
Source: J Pathol. 2017 Feb 24;241(5):600–13. doi: 10.1002/path.4864 (PMC5363360; doi:10.1002/path.4864)
Supplement: Supplementary file 1 — Supplementary discussion. Further discussion on other gene clusters of interest and justifications for the assignation of annotation of co‐expression signatures without a significant or relevant gene ontology term [file PATH-241-600-s001.docx]

# Supplementary Discussion

## Contaminating cell signatures

Several co-expression clusters were found in the RNA-seq dataset that are likely derived from contaminating cell types (Table S2, sheet 5). A skeletal muscle co-expression signature comprised of 76 transcripts and was primarily driven by high expression in a single sample was observed in the RNA-seq data. The cluster contained several skeletal muscle-specific myosins (*MYH1, MYH2*) and troponins (*TNNC2, TNNI1*), and GO enrichment analyses showed an over-representation of number of terms relating to striated muscles (e.g. GO:0006942, regulation of striated muscle contraction). The largest co-expression cluster in the RNA-seq data actually appeared to be derived from a contamination with testis. Cluster 1 contained 264 transcripts with many genes being associated with testis and spermatogenesis and enriched in GO terms relating to spermatids (e.g. GO:0007286, spermatid development). The genes in this co-expression cluster were expressed in a number of male suprapubic samples, although surprisingly they were also observed to be ‘expressed’ at low levels in number of male leg samples and in one female suprapubic sample. The formation of a neuronal co-expression cluster was driven by high expression in one individual (Cluster 43, 25 transcripts), which was also the case with glial cell co-expression although in a different individual (Cluster 83, 14 transcripts). The neurone co-expression cluster consisted of genes relating to synaptic processes (e.g. GO:0048489, synaptic vesicle transport), whilst the glial co-expression cluster consisted of genes relating to myelination (e.g. GO:0014015, positive regulation of gliogenesis). A co-expression cluster seemingly derived from pancreas (Cluster 29, 38 transcripts) was comprised largely of pancreatic digestive enzymes and as a cluster over-represented in GO terms including lipid digestion (GO:0044241), and a co-expression cluster seemingly derived from gastrointestinal mucosa was comprised largely of genes associated with this epithelium (Cluster 97, 12 transcripts) and over-represented in GO terms such as digestion (GO:0007586).

## Evidence to support annotation of *SkinSig* gene signatures

### Sebaceous gland

Sebaceous glands are small oil-producing glands, usually attached to hair follicles that release sebum into the follicular duct. The sebaceous gland *SkinSig* signature contains 105 genes. Gene set enrichment analysis suggested the signature to related to fatty acid metabolism (linolenic acid, monocarboxylic acid, oxoacid, triglyceride, cholesterol, sterol, steroid, fatty-acyl-CoA). Although this signature shares a number of GO terms also associated with the adipocyte signature, it lacks adipocyte marker genes, such as *ADIPOQ*, *AGPAT2, FABP4* and *LPL*. It does, however, contain multiple genes known to be expressed in human sebaceous glands, including *CIDEA* [39], *DGAT2* [40], and *FADS2*[41]. One of the markers for sebaceous glands, *MC5R* [42], is only found in the microarray dataset annotation and is therefore not included in the final signature list. The presence of other genes in the signature are supported by gene knockout experiments. *CIDEA* knockout mice show reduced sebum secretion [39], knockout mice of *ELOVL3* [43] and *FA2H* [44] demonstrate enlarged sebaceous glands, and transgenic mice with increased levels of *APOC1* demonstrate a reduced level of sebum production and sebaceous gland atrophy [45].

Connected to hair follicles, sebaceous glands secrete sebum onto the skin surface; disruption of this process may affect barrier functionality of the stratum corneum and hair growth [43, 44]. For instance, fatty acid analysis has suggested a shift in lipid composition resulted from *ELOV3* ablation, resulting in reduced water repulsion and increased trans-epidermal water loss [43].

### Eccrine Sweat gland

Eccrine glands are distributed across the human skin in large numbers, forming a thermoregulatory organ that primarily secretes electrolyte containing water. The 101 genes in this signature are enriched for the GO term, saliva secretion (GO:0046541, p=3.56E-02). Saliva secretion and sweat production may have shared mechanisms. For instance, hypohidrotic ectodermal dysplasia is characterised by reduced number of sweat glands and reduced saliva secretion; this condition has been associated with *EDAR* mutations [46]. The involvement of *CFTR* in sweat gland function is seen in cystic fibrosis; the disorder can be diagnosed through a sweat test as defects in *CFTR* results in faulty chloride reabsorption, leading to elevated salt content [47].

Other genes that have been previously been implicated in sweat glands arise from the knockout models for *FOXA1* and *AQP5*, the former abolishes the ability to sweat whilst the latter dramatically reduces the number of active sweat glands, despite continued sweat gland morphogenesis [48, 49]. *CFTR*, *SLC12A2* (previously known as *NKCC1*), *KRT8*, *AQP5* and Na+-K+ ATPase (*ATP1B1*) have previously been identified in the secretory portion of human sweat gland [50].

Several genes involved in tight junctions, ion channels, solute carriers that have not previously been reported to be involved in sweat glands are also present in this signature.

### Apocrine gland

The apocrine gland is primarily responsible for body odour (axillary odour). These glands secrete non-odorous precursor molecules onto the skin surface, where they are metabolized by bacteria and generate the volatile sulphur component present in human odour. Of the 25 genes in the signature, two genes have previously been implicated in apocrine glands: *ABCC11* [51] and *ACSM1* [52]. The involvement of *ABCC11* in apocrine glands was shown in individuals with a single nucleotide polymorphism of the gene (538G > A); the precursor molecules of the odorous components is almost undetectable in these individuals [51]. Expression of *ACSM1* in apocrine sweat gland has been demonstrated by immunohistochemistry and suggested as a marker for invasive apocrine carcinomas of the breast [52].

Other than these scant data are available on the genes expressed by these glands. Extramammary Paget’s disease, a rare cutaneous carcinoma, occurs preferentially at skin sites where apocrine sweat glands are found. The primary form of this disease is an in situ carcinoma of the apocrine gland ducts. Five of the 25 genes in this annotation (*ABCC11*, *CAPN13*, *CXCL17*, *DHRS2* and *KMO*) were amongst those upregulated by more than 3.5 fold in extra-mammary Paget’s disease. Twelve of the genes in the eccrine sweat gland signature reported in this study were also upregulated in extra-mammary Paget’s disease, suggesting there may be shared genes between the two types of sweat glands. For instance, *KRT7*, has been detected in high levels in a case of metastatic apocrine-gland carcinoma [53].

### Y-chromosome (male-specific)

Although not enriched in any GO terms, all 13 of the genes in this signature are located on the Y-chromosome and are only expressed in samples derived from male donors (Figure 2e).

### Keratinocyte (subset)

Immunohistochemistry sections from the Human Protein Atlas and literature suggest that genes in this annotation are found in the suprabasal region, with the strongest expression in stratum granulosum and no expression at stratum basale [54]. However, the exact function of this co-expression module is unclear, with studies supporting several roles for most genes, either relating to inflammatory response or concerning keratinocyte proliferation, migration or differentiation.

The majority of genes shows altered expression in inflammatory skin disorders, including *DEFB4A*, *SPRR2A*, *S100A7*, *S100A7A*, *S100A8*, and *S100A9*, and some have been shown to contribute to skin defence immunity [55]. *DEFB4A* has been demonstrated to be upregulated upon bacterial infections [56]. *S100A7* has been shown to have antimicrobial properties [57]. *S100A8* and *S100A9* are constitutively expressed by myeloid cells, but are also found in damaged skin or in inflammatory skin conditions [58].

However, in addition to their suggested involvement in inflammation, most genes in this signature are also involved in keratinocyte proliferation, migration and differentiation. Notably, immunosuppressed organ transplant recipients have increased risks of squamous cell carcinoma and overexpressed *S100A8* and *S100A9* [59]. *S100A8*/*9* heterodimer can induce keratinocyte proliferation and migration; this observation in immunosuppressed individuals suggests that their influence on keratinocytes may occur in the absence of inflammatory response. Furthermore, *S100A7* and *S100A9* have been shown to be upregulated in normal keratinocytes when cells are promoted to differentiate, and are found to be absent in undifferentiated basal cell carcinoma and strongly expressed in carcinoma, keratoacanthoma and differentiated squamous carcinoma [54]. *SPRR2A*, which encodes cornified envelope precursor proteins, has been linked to keratinocyte terminal differentiation [60], and its expression has been shown to be induced in response to epidermal injury.

##

## *Keratinocyte differentiation*

The epidermis can be divided into the stratum corneum, stratum spinosum, stratum granulosum and stratum basale. However, the co-expression networks of the two studies did not indicate definitive and consistent separation for genes associated with these different layers. This may in part due to the tightly regulated ratios between the different layers within normal skin. Co-expression analysis relies on differences between cell populations across samples, and if different cell layers are largely present in synchrony, genes specific for the individual layers would be considered to be co-expressed.

The 78 gene keratinocyte differentiation signature is likely to predominately reflect the suprabasal keratinocytes; of the 21 known suprabasal markers upregulated in the microarray analysis by Mattiuzzo et al. [91], 10 are found in the keratinocyte differentiation signature in this study. Genes in an unrelated co-expression cluster, labelled as keratinocyte (subset), appear to have a gradient-expression throughout the epidermis in immunohistochemistry images on the Human Protein Atlas repository, with the highest expression at stratum granulosum and mostly negative at stratum basale.

Another potential reason for the lack of the signature’s specificity for the individual epidermal layers is the dynamic range of the signal intensity in the marker genes. *KRT1*, *KRT5*, *KRT10*, *KRT14* are in the top 10 most highly expressed genes in the RNA-seq dataset, but not in the microarray dataset (Figure S1D). As *KRT5* and *KRT14* are specifically expressed in stratum basale, whilst *KRT1* and *KRT10* in the spinous and granular layers of the epidermis, the different dynamic range for highly abundant genes (Figure S1D) may contribute to the lack of detection of co-expression clusters specific for the different keratinocyte layers. For instance, there may be signal saturation for the microarray dataset or a non-linear relationship between these highly expressed genes and other genes unique to the differentiation states.

##

## Other clusters of interest

Co-expression of some cellular markers was seen as small clusters of genes that were consistent between both datasets, although not grouped with a larger set of markers. This may reflect the presence of these markers in multiple cell types, or that they have a non-linear relationship with other markers for the other markers of the particular cell type. These markers include markers for antigen presentation, which was in Cluster1518 (*CD1A*, *CD207*, *FCGBP;* Table S2, sheet 6) and Cluster0411 (*CD1A*, *CD207*, *FCGBP*, *HLA-DQB2;* Table S2, sheet 5) for the microarray and RNA-seq dataset. While *CD1A* and *CD207* had been used as common markers for Langerhan cells, these markers appear to cluster completely away from macrophage/DC markers and have a closer relevance to antigen presentation. However, the cluster size is too small to make a firm determination regarding their function.

Another group of co-expressed genes that is common between the two datasets comprised of *ATF3*, *DUSP1*, *FOS*, and *FOSB* in common, with *JUN*, *JUND*, *PPP1R15A*, *RN7SL512P*, and *RND3* in the RNA-seq dataset (Cluster0136; Table S2, sheet 5), and *ZFP36* in the microarray dataset (Cluster0483; Table S2, sheet 6). This co-expression cluster is significantly increased in skin challenged by a high dose of UV radiation in the dataset, GSE56754 [61, 62]. Using GO enrichment with all of the genes from the two studies, the top three most enriched terms were: “response to purine-containing compound”, “response to cAMP” and “response to calcium ion”. Literature search have suggested many of these genes to be involved in early response to DNA damage, such as UVR: *ATF3*, [63], *DUSP1* [64], *FOS* [65], *JUN* [66], and *JUND* [67]. Activator protein 1, composed of proteins belonging to the Fos and Jun families that *JUN*, *JUND*, *FOS* and *FOSB* are part of, has been shown to be involved in various aspects of the skin physiology [68].

Another cluster of interest is only present in the RNA-seq dataset, where *HOXB5*, *HOXB6* and *HOXB-AS3* (Cluster0839; Table S2, sheet 5) were co-expressed and demonstrated higher expression in the suprapubic region, consistent with reported restriction of expression of HOXB genes to fibroblasts of the trunk [69].
