# Supplementary material for: Derivation of marker gene signatures from human skin and their use in the interpretation of the transcriptional changes associated with dermatological disorders
Source: J Pathol. 2017 Feb 24;241(5):600–13. doi: 10.1002/path.4864 (PMC5363360; doi:10.1002/path.4864)
Supplement: Supplementary file 2 — Supplementary figure and table legends [file PATH-241-600-s002.docx]

**Supplementary figure and table legends**

**Figure S1. Sample–sample correlation and signal comparisons between the RNA-seq and microarray datasets.** **(A)** Sample–sample correlation plots of data used in these studies, using the maximum Pearson correlation coefficient threshold that still retained all samples for the RNA-seq (*r* ≥ 0.93) and **(B)** the microarray (*r* ≥ 0.97) datasets. There is minimal sample separation due to the main sample attribute, i.e. study (microarray) or site of sampling (RNA-seq). **(C)** Log-scale plot of median expression values, showing that expression levels for the majority of genes are positively correlated in the two datasets. **(D)** Of the top ten genes with the highest expression for the microarray (red) and RNA-seq (blue) datasets, there were only two genes in common (*RPS18* and *KRT14*). A higher dynamic range was also observed in the RNA-seq dataset.

**Figure S2. Pearson correlation thresholds in randomized and original data. (A)** When expression values for each gene were randomized across the samples of the RNA-seq dataset, only 95 pairing (edges) were observed at a threshold used in this analysis (*r* ≥ 0.73), whilst the untransformed data yielded 87 121 edges. Taking into account that a total of 123 802 980 calculations were made for every possible combination of gene–gene Pearson correlation analysis, the frequency of a pair of genes reaching the *r* threshold is 7.7 × 10^−7^ (blue), compared with a frequency of 7.0 × 10^−4^ for non-random correlations observed in the actual dataset (red). This supports the notion that the vast majority of relationships used to build the network analysed here are non-random. **(B)** Similar analysis was done to the microarray dataset. The randomized version of the dataset shows a frequency of 1 × 10^−8^ for a random correlation (blue) to occur at *r* ≥ 0.66, compared with a frequency of 6.7 × 10^−4^ observed in the actual dataset (red).

**Table S1. Details on the datasets used in this study**

The subject details, experimental design, data source, and comparisons for all datasets included in this study are listed in Table S1. This includes the three primary data datasets, 22 validation datasets for skin conditions, three psoriasis datasets for integrating the keratinocyte differentiation signature, and one ageing dataset.

**Table S2. *SkinSig* and co-expression signatures derived from the analysis of different datasets derived from normal human skin**

The supplementary file includes the co-expression signatures for all network analysis, including the individual full original datasets, gene-symbol restricted datasets, and *SkinSig*.

**Table S3. Gene ontology enrichment and example genes for each signature**

In addition to highlighting a number of key genes and significant gene ontology (GO) enrichment result for each *SkinSig*, the supplementary file also includes the full set results from gene ontology enrichment.

**Table S4. Comparison between *SkinSig* and genes found to be significantly altered during** **ageing**

Genes reported to change in their expression during ageing are compared with *SkinSig*, showing a high percentage of the sebaceous gland and hair signatures being reported to alter with ageing by Glass *et al* [19].
